# Association between enterovirus infection and clinical type 1 diabetes mellitus: systematic review and meta-analysis of observational studies

**DOI:** 10.1017/S0950268821002442

**Published:** 2021-12-10

**Authors:** Sen Yang, BiYing Zhao, Zhen Zhang, XiaoLin Dai, YiLi Zhang, LanWei Cui

**Affiliations:** 1 Department of Pediatrics, First affiliated Hospital of Harbin Medical University, YouZheng Street 23, Harbin 150001, China; 2 Department of Pediatrics, Children's Hospital of SooChow University, JingDe Road 303, SooChow 215002, China

**Keywords:** Clinical T1DM, enterovirus infection, meta-analysis, observational studies

## Abstract

Numerous animal models and epidemiological and observational studies have demonstrated that enterovirus (EV) infection could be involved in the development of clinical type 1 diabetes mellitus (T1DM), but its aetiology is not fully understood. Therefore, we reviewed the association between EV infection and clinical T1DM. We searched PubMed and Embase from inception to April 2021 and reference lists of included studies without any language restrictions in only human studies. The correlation between EV infection and clinical T1DM was calculated as the pooled odds ratio (OR) and 95% confidence intervals (CIs), analysed using random-effects models. Subgroup and sensitivity analyses were performed to evaluate the robustness of the associations. A total of 25 articles (22 case–control studies and three nested case–control studies) met the inclusion criterion including 4854 participants (2948 cases and 1906 controls) with a high level of statistical heterogeneity (*I*^2^ = 80%, *P* < 0.001) mainly attributable to methods of EV detection, study type, age distribution, source of EV sample and control subjects. Meta-analysis showed a significant association between EV infection and clinical T1DM (OR 5.75, 95% CI 3.61–9.61). There is a clinically significant association between clinical T1DM and EV infection.

## Introduction

Type 1 diabetes mellitus (T1DM) is generally believed to be a chronic autoimmune disease characterised by the destruction of insulin-producing *β*-cells that results from a complex interaction between genetic susceptibility, immunological factors and environmental agents [1]. It has been reported that the present global number of individuals with diabetes was estimated at 415 million, but has reached as much as 642 million by 2040. The estimated incidence rates of T1DM increased annually by 1.4% during 2002–2012 in America [2], 1.01% during 2010–2015 in China respectively [3, 4]. The rapid increase in incidence, especially in children under the age of 5 years [5], cannot be fully attributed to genetic factors. Reports have linked viral infections [6], obesity [7], socioeconomic status [8], vitamin D deficiency [9, 10], diet, immunisation, seasonal variation [11] to an increased risk of T1DM. However more recent evidence regarding a putative role for enterovirus (EV) infection in the development of clinical T1DM comes from case–control studies that have shown a significant temporal association after enterovirus epidemics, and the detection of EV RNA or EV capsid protein in pancreatic biopsies of patients with current onset T1DM [6]. On the other hand, evidence from diabetic animal models and cell studies suggests that EVs are likely to destroy the pancreas via immunological cross-reaction (molecular mimicry) because of the sequence homology between the coxsackievirus P2 protein and glutamic acid decarboxylase 65 (GAD65) or is directed to destroy insulin-producing islet cells via T lymphocytes (bystander damage) [1]. T1DM may also contribute to the children's psychological and mental problems [12], such as depression and anxiety, since a strong correlation between diabetes and the status of mind or quality of life in children has been reported. Besides, diabetes could increase the economic burden to families and societies in low- and middle-income countries because of lifelong treatment and management of illness. Furthermore, identification of these risk factors could lead to a better understanding of T1DM and contribute to developing strategies to prevent T1DM so as to reduce the economic burden of diabetes and improve the quality of life.

In 2004, a systematic review of coxsackie B virus serology did not indicate a relation with T1DM [13], but there was another study showing that EV infection, confirmed only by reverse transcription-polymerase chain reaction (RT-PCR), did show a clinically significant association with T1DM in 2011 [14]. Moreover, these case–control or cohort studies did not increase the statistical power and provided precise estimates because of the relatively small sample size for each individual study. However, the correlation between EV and clinical T1DM remains unclear due to the source of EV samples, different methods to confirm EV infection and study type and so on. Based on these facts, we conducted a systematic review and meta-analysis to clarify the relationship between EV infection and the risk of clinical T1DM.

## Methods

This study was performed in accordance with the Meta-analysis Of Observational Studies in Epidemiology (MOOSE) [15] and was registered on PROSPERO (registration number: CRD42021236044).

### Search strategy

Two reviewers (SY and XL D) independently performed a systematic search for observational studies of enterovirus (EV) infection and clinical T1DM according to Medical Subject Headings (MeSH) or Emtree combined with free-text terms on PubMed and Embase, from inception to April, 2021. The search terms used were ‘type 1 diabetes’, ‘enterovirus infection’, ‘echovirus infection’ and ‘coxsackievirus infection’. The search strategy is reported in detail in the Supplementary Materials. The search was confined to original articles including humans in any language and was conducted by manually searching the reference lists of the eligible studies and by direct contact with authors.

### Study selection criteria

Studies were eligible for inclusion in line with the below criteria: (1) a case–control or cohort study design; (2) assessment of the relation between EV infection and clinical T1DM; (3) established or newly diagnosed T1DM without HIV or hepatitis viruses; (4) evidence of EV infection via measuring virus RNA or specific antibodies in blood, stool or tissue of patients or laboratory investigations or other biopsies; (5) available data on the odds ratio (OR) with 95% confidence interval (CI) or numbers of events/total in both case and control; (6) non-human studies were excluded. Disagreements between the two reviewers were resolved via discussion with a third arbitrary investigator (LW C), when necessary.

### Data extraction

The information of each study were extracted using a standardised form as follows: first author's name, date of publication, design of the study, location, age distribution, number of cases and controls (matching criteria), methods to confirm EV infection, virus species or serotypes, assessment of diabetes and islet autoantibodies. One investigator (ZZ) extracted above the data checked by another investigator (YL Z).

### Quality assessment

Two investigators (SY and BY Z) independently evaluated the included study quality independently using the Newcastle-Ottawa scale (NOS) for case–control studies or cohort studies, as recommended by the Cochrane Collaboration, and different opinions were resolved through consensus. This tool assessed three areas-selection, comparability, exposure or outcome-total score of 9 stars, with 5 stars or more deemed as good methods. In the comparability category, we highlighted the evaluation of controls matched for age and sampling time, as these two factors are most likely to affect the incidence of EV infection. The possibility of publication bias was evaluated by visual inspection of the funnel plot.

### Statistical analysis

Review Manager software (version 5.3) was used to calculate pooled ORs with 95% CI and *P* value for EVs infection in patients with diabetes *vs.* no diabetes from the published data in studies using the Mantel–Haenszel method. We analysed the association between EV infection and clinical T1DM using both fixed and random-effects models. However, only combined ORs from the random-effects models are presented because of a high level of heterogeneity in the study design. A forest plot summarised the results of all eligible studies. Statistical heterogeneity was explored using Cochran's Q and *I*^2^ statistics, indicating the proportion of variance in outcomes between studies. Statistical significance was defined as *P* < 0.05 was considered statistically significant heterogeneity, while *I*^2^ less than 25%, 50% and 75% were regarded as low, moderate and high heterogeneity respectively. Subgroup and sensitivity analyses were conducted for age distribution, the initial time of clinical T1DM, methods to confirm EV infection, source of EV sample and control subjects, virus species or serotypes, study type and summary ORs were calculated. We also performed sensitivity analyses by individual study, study size, study location and NOS score.

## Results

### Study selection

**Fig. 1.**
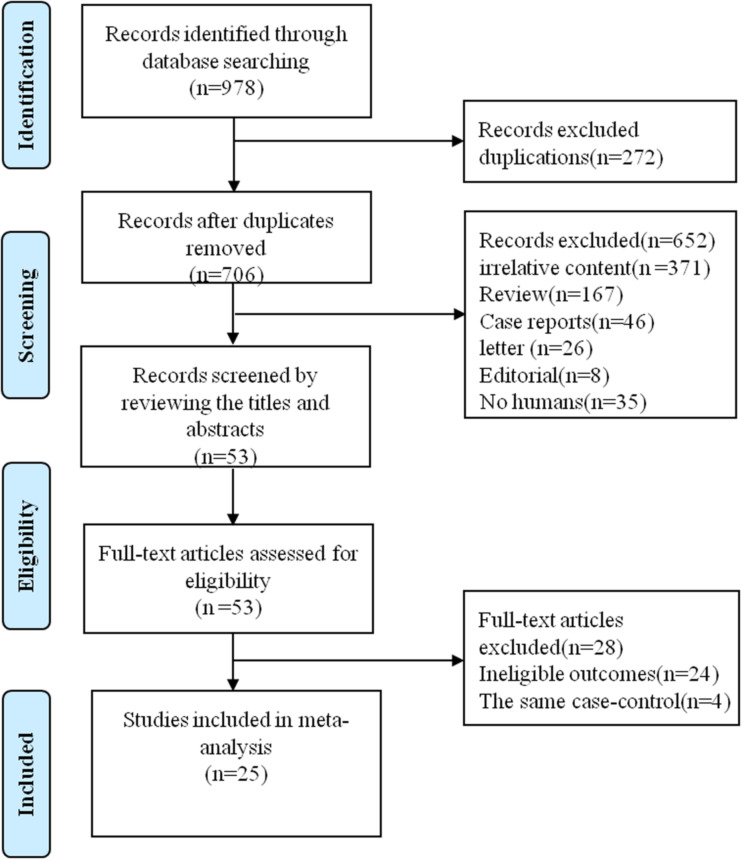
Flow diagram of the literature selection process.

Our search returned all 706 publications after the removal of 272 duplicate articles. We identified and included 53 potential original articles by screening titles and abstracts. Furthermore, 25 relevant studies were evaluated by reviewing the full text and finally included in the systematic review and meta-analysis. Four studies were excluded owing to the same case–control subjects for different research aims. Figure 1 shows the search flowchart for eligible studies.

### Characteristics of included studies

Demographic characteristics of the included 25 studies are presented in Table 1. All studies were case–control designs, and of these samples of three nested case–control studies were collected from diabetic cohort studies. The majority of included study subjects were from Europe and a few participants were from non-European countries. Twenty-five studies included 2150 patients with T1DM and 2704 controls, aged range between 0 and 70 years, but who were mostly children, adolescents and young adults. EV infection was confirmed by real RT-PCR and *in situ* hybridisation (ISH) to detect EV-RNA in 15 studies, specific IgM antibodies using neutralisation test (NT), ELISA, immunofluorescence assay (IFA) and radioimmunoassay (RIA) to identify antibodies against EV or coxsackievirus for seven studies, and immunohistochemistry (IHC) to investigate enteroviral capsid protein vp1 for three studies. Of the 25 studies, five studies simultaneously used two methods of EV detection. Most articles did not report data on EV species or serotypes, but only five studies provided data on IgM antibodies against coxsackievirus B serotypes and of which one only examined echovirus and coxsackievirus A.

**Table 1.** Summary of an individual study investigating type 1 diabetes and enterovirus infection

Study	Country	Cases/Controls	Age (years)	Details of cases	Details of controls	Method of detection	Details of methods to confirm viral infections	Islet Autoantibody	NOS score
Antonio, 1985 [16]	Italy	22/46	0~16	T1DM onset; Samples were collected from 1981 to 1982	place-matched healthy children in the same season	NT	CB1~6 neutralising antibodies in serum		7
Gun, 1985 [17]	Sweden	24/48	2~15	T1DM onset, Samples were continuously collected from 1982 to 1984	48 age, sample time-matched nondiabetic children in 24 of which have non-EV infections; the other 24 for planned surgical procedure	RIA	CB-virus-specific IgM in serum		8
Donn, 1992 [18]	America	225/163	0~29	T1DM onset; HLA-DR3 typing; Samples were collected from 1984 to 1987	age, sex, sample time and socioeconomic status-matched friends	NT	CB-virus-specific IgM in serum		8
Frisk, 1992 [19]	Sweden	35/47	case:0~15;control:3~18	T1DM onset;Samples were collected from 1983-8	siblings	RIA	CB-virus-specific IgM in serum		7
Nairn, 1999 [20]	British	110/182	1~17	T1DM onset;Samples were collected from 1991-7	age,place,sample time-mactched nondiabetic children	RT-PCR	EV-RNA in serum		8
Lonnrot, 2000 [21]	Finland	47/34	2.5~20	T1DM onset;Samples were collected from Finland DiMe study	siblings	RT-PCR	EV-RNA in serum	ICA,IAA,GAD,IA-2	7
Miroslav, 2000 [11]	Slovak	336/707	children	T1DM onset; Samples were collected from 1978-91; cases and controls were taken from the Slovak National Register of Childhood Diabetes	age-matched healthy children were collected from 1985-98	NT	CB antibody in serum		5
Wassim, 2000 [22]	Sweden	56/24	case:3~69,control:7~66	12 newly T1DM children and 20 newly T1DM adults;13 previously T1DM children and 13 previously T1DM adults; Samples were collected from 1997 to 1998	17 children and 20 adults	RT-PCR	EV-RNA in serum		8
Maria, 2003 [23]	Austrilia	206/160	case:0.7~15.7 control:0.5~15.8	T1DM onset,HLA-DRB/DQB typing;Samples were collected from 1997–1999	children from the community	RT-PCR	EV-RNA in either plasma or stool	ICA,IAA,GAD-65,IA-2	9
Moya, 2005 [24]	Germany	47/100	case:11~15 control:10~16	T1DM onset	autoantibody-positive and negative children for 50,50 respectively	RT-PCR/ELISA	EV-RNA and CB-virus-specific IgM/IgG in serum	GAD-65,ICA,IA-2	7
Francesco, 2007 [6]	Italy	6/26	case:4~26 control:14~53	T1DM onset; 5 multiorgan donors and 1 whole pancreas graft	normal multiorgan donors	IHC	enteroviral VP-1 in pancreatic tissues		6
Elfaitouri, 2007 [25]	Sweden	33/51	0~17	T1DM onset;Samples were collected from 2000–1	24 siblings and 27 healthy children	RT-PCR	EV-RNA in PBMCs	GAD-65	7
Lius, 2007 [26]	Cuba	34/257	case:1~15 control:1~47	T1DM onset	32 ICA positive relative controls;31 ICA negative relative controls;194 healthy subjects,age,sex,place,sample time-mactched	RT-PCR	EV-RNA in serum	ICA	6
Oikarinen, 2008 [27]	Finland	12/10	case:18~53 control:23~71	0~50 duration of T1DM;Samples were collected from 1995–2000	non-diabetic subjects	ISH and IHC	enterovirus in intestinal musoca		7
Richardson, 2009 [28]	British	72/119	case:1~42 control:23~71	T1DM onset	11 normal neonate;39 normal children;69 normal adults	IHC	enteroviral capsid protein vp1 staining in pancreatic tissue		7
Barbara, 2010 [29]	The Netherlands	10/20	case:5~14 control:6~17	T1DM onset; HLA typing; Samples were collected from 2003–4	hospitalised children without endocrine disorders; HLA typing	RT-PCR	EV-RNA in PBMCs and plasma		5
Mercalli, 2012 [30]	Italy	25/48	case:3~77 control:1~67	T1DM duration:1~57;Samples were collected from 2005–6	27 healthy individuals and 21 patients with ceoliac disease	ISH/RT-PCR/IHC	EV-RNA/VP-1 in intestinal musoca	GADA,IA-2A,IAA,TGG,TGA,TMA	7
Maarit, 2012 [31]	Finland	39/81	case:18~63 control:18~76	T1DM duration:0~38, HLA-DR typing;Samples were collected from 1995–2000	40 coeliac disease patients and 41 nondiabetic subjects	ISH/RT-PCR /IHC	EV-RNA and VP1 protein in intestinal mucosa		7
Salvatoni, 2013 [32]	Italy	24/116	case:6~13.6 control:4.9~46	T1DM onset;Samples were collected from 2010–2	20 sibilings,41 parents,29 non-diabetic children and 26 healthy adults;age,place,sample time-matched	RT-PCR	EV-RNA in plasma	GADA,IA-2,IAA,ZnT8	7
Sami, 2014 [33]	European	249/249	case:1.1~22.7 control:1.0~23.5	T1DM onset;HLA-DR3/DR4 genotype;cases and controls were taken from European VirDiab Study	age,sex,place and sampling time-matched children	NT	CB Antibodies in serum	ICA,IAA,GADA,IA-2A	8
Cekin, 2014 [34]	Turkey	86/100	9.9 ± 2.3	24 T1DM onset;62 previously T1DM	age,sex-matched children	RT-PCR/NT	EV-RNA and CB4 antibody in plasma	GAD	7
Imen, 2017 [35]	Tunisia	95/141	1~48	T1DM duration:0~30,41 children and 54 adults	57 children and 84 adults	RT-PCR	EV-RNA in plasma	GAD	6
Waled, 2018 [36]	Egypt	382/100	case:2~16 control:3~14	T1DM;Samples were collected from 2013–4	sex,age-matched children	RT-PCR	EV-RNA in serum		6
Giovanni, 2018 [10]	Italy	82/117	2.1~18	T1DM	sex, age, place and sample time-matched short stature or minor trauma children	RT-PCR	EV-RNA in serum		7
Murat, 2018 [37]	Turkey	40/30	1~16	T1DM onset	sample time and place-matched healthy children	IFA	IgM Antibodies to EV in serum	GADA,ICA,IAA	7

T1DM, type 1 diabetes mellitus; NT, neutralisation test; RIA, radioimmunoassay; IFA, immunofluorescence assay; IHC, immunohistochemistry; RT-PCR, reverse transcription-polymerase chain reaction; CB, group B coxsackievirus; EV, enterovirus.

### Meta-analysis results

**Fig. 2.**
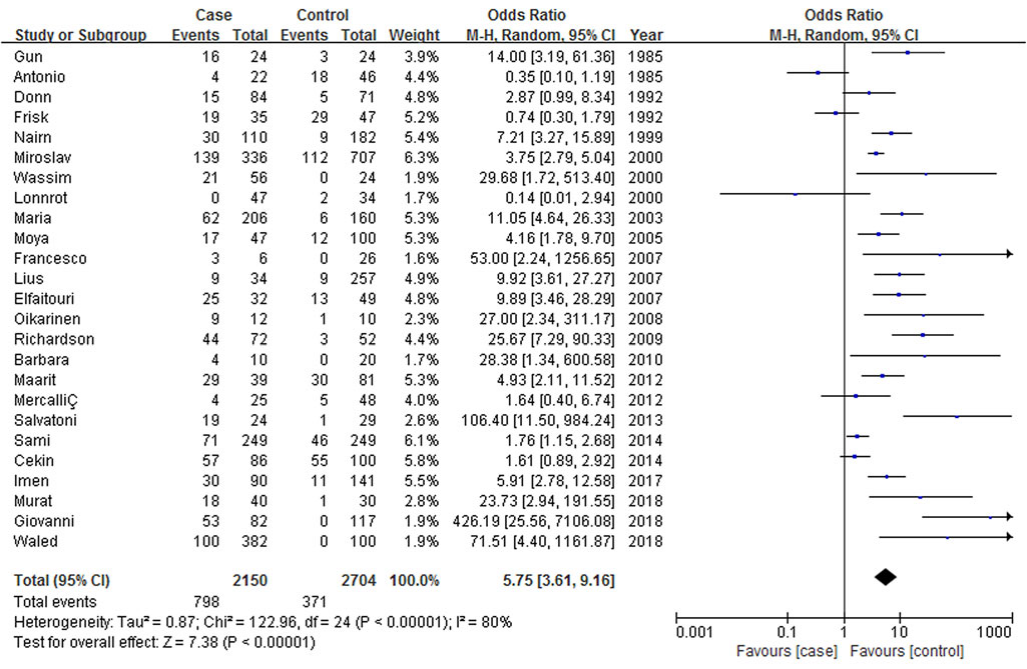
Forrest plot of the association between clinical T1DM and EV infection.

The results suggested that EV infection was significantly related to clinical T1DM mellitus as compared with no T1DM, but with evidence of high heterogeneity between the 25 studies (*P* < 0.001, *I*^2^ = 80%) (Fig. 2). ORs ranged from 0.14 to 426.19, with a combined OR of 5.75 (95% CI 3.61~9.16)(Fig. 2).

### Subgroup and sensitivity analyses

We also performed subgroup analyses with respect to methods to confirm EV infection, source of EV sample, virus species or serotypes, initial time of clinical T1DM, age distribution, source of control subjects and study type (Table 2). The combined ORs for NT, RIA, ISH, IHC and RT-PCR were 1.58 (95% CI 0.76–3.30), 3.02 (95% CI 0.17–56.64), 5.21 (95% CI 2.31–11.79), 7.29 (95% CI 1.42–37.58), 7.48 (95% CI 4.20–13.32) respectively. There was no heterogeneity (*I*^2^ = 0%, *P* = 0.35)across the two studies that measured EV-RNA in the intestinal mucosa by ISH, but the other subgroups by NT, RIA, IHC and RT-PCR showed significant heterogeneity (Table 2).

**Table 2.** Summary odds ratios and heterogeneity for an association of EV infection and clinical T1DM in subgroup and sensitivity analyses

Variables	No. of studies	OR (95% CI)	Weight (%)	*I*^2^%	*P* for heterogeneity
Geographical areas					
Europe	18	5.72 (2.94~11.14)	70.4	83	<0.001
Non- Europe	7	6.02 (3.02~12.01)	29.6	75	<0.001
Age distribution (years)					
0~9	2	33.82 (1.87~612.91)	6.4	74	<0.001
0~20	14	4.89 (2.51~9.51)	52.9	83	<0.001
0~71	11	7.53 (3.61~15.72)	40.7	81	<0.001
The initial time of clinical T1DM					
Newly clinical T1DM	18	4.76 (2.84~7.98)	81.9	82	<0.001
Previously clinical T1DM	4	4.91 (2.49~9.67)	18.1	32	0.22
Methods of EV detection					
NT	5	1.58 (0.76~3.30)	25.1	89	<0.001
RIA	2	3.02 (0.17~54.64)	8.4	91	<0.001
ISH	2	5.21 (2.31~11.79)	6.5	0	0.350
IHC	5	7.29 (1.42~37.58)	16	81	<0.001
RT-PCR	13	7.48 (4.20~13.32)	44	63	0.002
Source of EV sample					
Serum	14	3.90 (3.24~4.70)	73.3	83	<0.001
Intestinal Musoca	3	4.36 (2.24~8.49)	5.2	50	0.14
Plasma	5	4.49 (3.12~6.46)	19.1	77	0.002
PBMC	2	11.42 (4.27~30.58)	1.5	0	0.52
Pancretic Tissue	2	27.60 (8.48~89.78)	0.9	0	0.67
Type of controls					
Relatives	3	3.19 (1.65~6.14)	31.6	0	0.75
Normal Subjects	3	56.41 (3.54~899.49)	27.5	87	<0.001
Autoantibody positive individuals	2	1.07 (0.43~2.67)	21.5	40	0.20
Autoantibody negative individuals	2	2.29 (0.15~35.84)	19.4	83	0.01
Coxsackievirus Serotypes					
CB1	4	1.76 (1.18~2.63)	14.8	0	0.67
CB2	4	0.90 (0.50~1.64)	19	25	0.26
CB3	4	0.95 (0.68~1.33)	18.5	0	0.51
CB4	5	2.03 (0.87~4.75)	20.9	60	0.04
CB5	4	0.91 (0.37~2.21)	17.4	43	0.15
CB6	2	0.88 (0.50~1.53)	9.3	0	0.87
Sample size					
<100	12	5.88 (3.46~9.99)	57.7	83	<0.001
>100	13	6.00 (2.21~16.26)	42.3	80	<0.001
NOS score					
9~10	6	5.89 (2.42~14.38)	27.4	81	<0.001
8	13	5.12 (2.10~12.49)	50.6	85	<0.001
6~7	6	7.93 (3.75~16.78)	22.0	61	0.02
Study type					
CC	22	7.49 (4.20~13.36)	85.9	80	<0.001
NCC	3	2.16 (0.95~4.92)	14.1	83	<0.001

T1DM, type 1 diabetes mellitus; NT, neutralisation test; RIA, radioimmunoassay; IFA, immunofluorescence assay; IHC, immunohistochemistry; RT-PCR, reverse transcription-polymerase chain reaction; CB, group B coxsackievirus; CC, case–control study; NCC, nested case–control study.

When we analysed the source of EV sample, the summary ORs for serum, intestinal mucosa, plasma, peripheral blood mononuclear cells (PBMCs) and pancreatic tissue were 3.90 (95% CI 3.24–4.70), 4.36 (95% CI 2.24–8.49), 4.49 (95% CI 3.12–6.46), 11.42 (95% CI 4.27–30.58), 27.60 (95% CI  8.48–89.78) respectively, with significantly statistically heterogeneity (*I*^2^ = 72.30%, *P* = 0.006) across the 5 groups, while there was no heterogeneity in PBMCs group (*I*^2^ = 0%, *P* = 0.52) and pancreatic tissue group (*I*^2^ = 0%, *P* = 0.67), moderate heterogeneous in intestinal mucosa group (*I*^2^ = 50%, *P* = 0.14), high heterogeneous in serum group (*I*^2^ = 83%, *P* < 0.001) and plasma group (*I*^2^ = 77%, *P* = 0.002)respectively (Table 2).

For only six studies that examined specific IgM antibodies to coxsackievirus B (CB) serotypes, the pooled ORs for CB1, CB2, CB3, CB5, CB6 were 1.76 (95% CI 1.18–2.63), 0.90 (95% CI 0.50–1.64), 0.95 (95% CI 0.68–1.33), 0.91 (95% CI 0.37–2.21), 0.88 (95% CI 0.50–1.53) respectively, while the pooled OR for CB4 was comparatively higher (2.03 (95% CI 0.87~4.75)) for the other CB1, CB2, CB3, CB5, CB6 serotypes and with moderate heterogeneity (*I*^2^ = 60%, *P* = 0.04) *vs.* no or mild heterogeneity respectively (*I*^2^ = 0%, *P* = 0.67; *I*^2^ = 25%, *P* = 0.26; *I*^2^ = 0%, *P* = 0.51; *I*^2^ = 43%, *P* = 0.15; *I*^2^ = 0%, *P* = 0.87) (Table 2).

The combined ORs for newly diagnosed clinical T1DM and previously diagnosed clinical T1DM were 4.76 (95% CI 2.84–7.98), 4.91 (95% CI 2.49–9.67), with high heterogeneity (*I*^2^ = 82%, *P* < 0.001) *vs.* mild heterogeneity (*I*^2^ = 32%, *P* = 0.22) respectively (Table 2).

For the summary, OR of age 0~9 years group (33.82 (95% CI 1.87–612.91)) was significantly higher in the 0~20 years group (4.89 (95% CI 2.51–9.51)) and age 0~71 years group (7.53 (95% CI 3.61–15.72)), probably because of the high rates of EV infection in children [18, 21, 34]. It is generally believed to children with the immature immune system in whom they have a greater risk of infection in comparison with adults who have a mature immune system. The rates background refers to this. Although there was no appropriate data on HLA genotypes in all cases and controls to perform subgroup analysis, we investigated the relationship between EV infection and clinical T1DM affected by HLA typing based on different sources of control subjects, The relative controls are selected from the siblings, while the normal subjects are collected from the unrelated individuals, so that we are able to elucidate the effect of a genetic factor on the result. The combined OR of relative controls (3.19 (95% CI 1.65–6.14)) was significantly higher for the normal (Table 2) subjects (56.41 (95% CI 3.54–899.40)), indirectly demonstrating that EV infection could increase the risk of clinical T1DM in genetically susceptible individuals (Table 2).

When we analysed the results from the only three nested case–control studies, the summary OR (2.16 (95% CI 0.95–4.92))was lower than that of the 22 case–control studies (7.49 (95% CI 4.20–13.36)), probably because of the variance in study design (Table 2). In summary, subgroup analyses indicate that none of the subsets significantly affected the stability of overall results, in addition to group B coxsackievirus that obviously decreased the combined OR of 5.75 (95% CI 3.61–9.16) for a relation between EV infection and clinical T1DM to that of 1.14 (95% CI 0.92–1.41) for an association between CB infection and clinical T1DM.

We also carried out a sensitivity analysis by study size, study location and NOS score to examine the robustness of the correlation. The summary ORs for more than 100 participants and less than 100 participants were 5.88 (95% CI 3.46–9.99), 6.00 (95% CI 2.21–16.26) respectively, with no heterogeneity between the groups. The pooled ORs for the European area and non-European area were 5.72 (95% CI 2.94–11.94), 6.02 (95% CI 3.02–12.01) respectively, with no heterogeneity between both groups. Sensitivity analysis by study quality was classified into three groups (8~9 score group, 7 score group, 5~6 score group) because all studies scored more than 5 on the NOS (Table 2). Although most studies did not report diagnostic criteria in detail for clinical T1DM, and clinical presentation and laboratory investigations were poorly described, insulin therapy was started in patients with T1DM after diagnosis. Finally, sensitivity analysis by individual studies did not significantly affect the summary effect estimates.

### Quality assessment

**Fig. 3.**
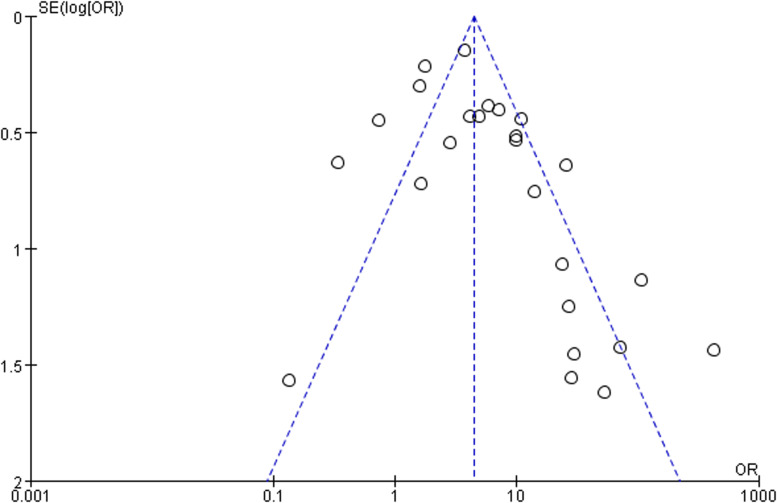
Funnel plot of the association between clinical T1DM and EV infection.

Newcastle Ottawa scores ranged from 5 to 9 stars, with all studies scoring 5 stars or more, suggesting good methodological quality overall, with no studies reporting a non-response rate (Table 1). The funnel plot showed reasonable symmetry, with no evidence of publication bias (Fig. 3). However there was great variability across the studies, with significant statistical heterogeneity; therefore, the meta-analysis results should be interpreted with caution when extended to the general population.

## Discussion

To our knowledge, this meta-analysis and systematic review are the first to report the relationship between EV infection and the risk of clinical T1DM by systematically reviewing molecular and serological observational studies. This study, which included 25 articles, suggest that EV infection had more than about six times the risk of clinical T1DM, approximate 34 times the risk in children when compared with the control. Those who have clinical TIDM with positivity for islet autoantibodies are not at greater risk of EV infection than those with negative results for islet autoantibodies; therefore, so enterovirus infection might be a risk factor for clinical type 1 diabetes. Our results suggest that the pathogenesis of clinical T1DM triggered by EV may not be one mechanism. Subgroup analysis demonstrated that age group, methods of EV detection, source of EV sample, study design and genetic factors may have a tremendous influence on the results.

To date, a great many epidemiological and observational studies have investigated the relationship between EV infection and the risk of T1DM [6, 10, 11, 17, 20, 22–29, 31–33, 35–37]. However, their findings have been inconsistent because the prevalence and incidence of T1DM differ greatly for geographic areas, methods of EV detection, age and source of EV samples and genetically predisposed individuals. Since J. E. Banatvala *et al*. first reported evidence of the association between Coxsackie B1–5 viruses and children under the age of 5 years with clinical T1DM in Austria, England and Australia [38], subsequently most studies have been conducted in Europe. Overall, it is recognised by most investigators that EV infection could accelerate the progression of T1DM or transiently emerge autoantibodies associated with T1DM in genetically susceptible populations, however, we perform an analysis of the islet autoantibody-positive individuals due to lack of sufficient data that provided unreliable results. We included children and adults with clinical T1DM, decreasing the high rates background of bacterial and viral infections in children. Global studies were included to decrease the risk of geographical bias associated with infection rates. Most studies, however, were from European countries [6, 10, 16–26, 28, 30–33] where the incidence of diabetes is higher than that in Asian and African countries. Given the heterogeneity of study populations' heterogeneity, complex pathogenesis and multiple environmental agents, we used random-effects models due to high heterogeneity across individual studies, providing more conservative and reliable effect estimates. However, our results should be carefully interpreted due to significant statistical differences among all studies, when particular, as extended to the external population.

Although the initial factors of the anti-islet cell autoimmune response are not understood, a few possible mechanisms for the relationship between EV infection and risk of T1DM have been inferred. First, patients with clinical T1DM are more likely to be infected with a variety of pathogens, such as bacteria, viruses and fungi, compared to individuals without T1DM. Viruses can promote diabetes either by directly infecting and destroying islet beta cells or by triggering an autoimmune attack on islet cells [1]. In addition, seasonal variation plays an important role in the pathogenesis of T1DM [11]. Furthermore, diet and perinatal factors are more likely to increase the risk of developing T1DM [39]. Secondly, there was one possible fact that molecular mimicry due to homology between Glutamic acid decarboxylase 65 (GAD65) and a causative agent such as Coxsackie B4 virus. A search has investigated Several autoantigens (IAA, ICA, IA-2A and ZnT8) within pancreatic beta cells play significant roles in the initiation or progression of autoimmune pancreatic injury. Nonetheless, children or adults with another autoimmunity, most commonly autoimmune thyroiditis and coeliac disease, are at increased risk for catching T1DM; however, there was an extreme lack of available data in the eligible studies, so that we could not perform the related analysis. In the long term, clinical T1DM is autoimmunity that gives rise to a complex interaction between genetically susceptible populations and environmental factors [1].

In the future, larger multicentre international prospective or birth cohort studies could investigate the relationships between clinical T1DM and age distribution, genetic factors, enterovirus various and EV serotypes. Moreover, clinical trials are needed to develop useful and feasible strategies, as vaccines against EV species or serotypes, to lessen the prevalence and incidence of T1DM.

### Limitations

We performed a set of standard and comprehensive literature searches, and made no language restrictions to limit our ability to evaluate the association between EVs infection and the risk of T1DM. However, there were several limitations in our meta-analysis. First, the included studies were confined to case–control studies with inherent factors, such as different data collection, various detection methods of viral infections (RT-PCR, specific antiviral neutralising antibodies and hybridisation, and samples from different sites. Second, it was true that we performed subgroup and sensitivity analyses to reduce potential confounding factors, but all eligible studies also had a high level of heterogeneity. Third, other environmental agents might alter the risk of T1DM, such as maternal virus infection [40], cow's milk and vitamin D [41], moreover, it is impossible to improve all of these potential confounders in retrospective studies. Fourth, the results of this meta-analysis cannot prove that EV infection has a cause-and-effect role in the development of clinical T1DM. Despite these limitations, this meta-analysis has increased the statistical power by pooling the findings of a single case–control study with overall good methodological quality, to some extent, which was sufficient evidence to draw this conclusion.

In summary, we demonstrated that EV infection may be a dependent risk factor for clinical T1DM. Further studies are needed to explore the potential pathways and focus on whether the virus vaccine could decrease the risk of clinical T1DM or not.

## Data Availability

The data that support the findings of this study are available as Supplementary Materials.

## Supplementary material

For supplementary material accompanying this paper visit http://doi.org/10.1017/S0950268821002442. click here to view supplementary material
