# Supplementary material for: Association between enterovirus infection and clinical type 1 diabetes mellitus: systematic review and meta-analysis of observational studies
Source: Epidemiol Infect. 2021 Dec 10;150:e23. doi: 10.1017/S0950268821002442 (PMC8851353; doi:10.1017/S0950268821002442)
Supplement: Supplementary file 1 [file S0950268821002442sup001.docx]

**Supplementary Materials**

**Filters by humans**

**#1** "Diabetes Mellitus, Type 1"[Mesh]

**#2** ((((((((((((((((((((((((((Diabetes Mellitus, Insulin-Dependent[Title/Abstract]) OR (Diabetes Mellitus, Insulin Dependent[Title/Abstract])) OR (Insulin-Dependent Diabetes Mellitus[Title/Abstract])) OR (Diabetes Mellitus, Juvenile-Onset[Title/Abstract])) OR (Diabetes Mellitus, Juvenile Onset[Title/Abstract])) OR (Juvenile-Onset Diabetes Mellitus[Title/Abstract])) OR (IDDM[Title/Abstract])) OR (Juvenile-Onset Diabetes[Title/Abstract])) OR (Diabetes, Juvenile-Onset[Title/Abstract])) OR (Juvenile Onset Diabetes[Title/Abstract])) OR (Diabetes Mellitus, Sudden-Onset[Title/Abstract])) OR (Diabetes Mellitus, Sudden Onset[Title/Abstract])) OR (Sudden-Onset Diabetes Mellitus[Title/Abstract])) OR (Type 1 Diabetes Mellitus[Title/Abstract])) OR (Diabetes Mellitus, Insulin-Dependent, 1[Title/Abstract])) OR (Insulin-Dependent Diabetes Mellitus 1[Title/Abstract])) OR (Insulin Dependent Diabetes Mellitus 1[Title/Abstract])) OR (Type 1 Diabetes[Title/Abstract])) OR (Diabetes, Type 1[Title/Abstract])) OR (Diabetes Mellitus, Type I[Title/Abstract])) OR (Diabetes, Autoimmune[Title/Abstract])) OR (Autoimmune Diabetes[Title/Abstract])) OR (Diabetes Mellitus, Brittle[Title/Abstract])) OR (Brittle Diabetes Mellitus[Title/Abstract])) OR (Diabetes Mellitus, Ketosis-Prone[Title/Abstract])) OR (Diabetes Mellitus, Ketosis Prone[Title/Abstract])) OR (Ketosis-Prone Diabetes Mellitus[Title/Abstract])

**#3 #1 OR #2**

**#4** "Enterovirus Infections"[Mesh]

**#5** ((Infections, Enterovirus[Title/Abstract]) OR (Enterovirus Infection[Title/Abstract])) OR (Infection, Enterovirus[Title/Abstract])

**#6 #4 OR #5**

**#7** "Coxsackievirus Infections"[Mesh]

**#8** ((((Infections, Coxsackie Virus[Title/Abstract]) OR (Coxsackie Virus Infection[Title/Abstract])) OR (Infections, Coxsackievirus[Title/Abstract])) OR (Coxsackievirus Infection[Title/Abstract])) OR (Coxsackie Virus Infections[Title/Abstract])

**#9 #7 OR #8**

**#10** "Echovirus Infections"[Mesh]

**#11** ((((((Echo Virus Infections[Title/Abstract]) OR (Echo Virus Infection[Title/Abstract])) OR (Infection, Echo Virus[Title/Abstract])) OR (Infections, Echo Virus[Title/Abstract])) OR (Infections, Echovirus[Title/Abstract])) OR (Echovirus Infection[Title/Abstract])) OR (Infection, Echovirus[Title/Abstract])

**#12 #10 OR #11**

**#13 #6 OR #9 OR #12**

**#14 #3 AND #13**

**Embase search history**

**#14. #10 AND #13**

**#13. #11 OR #12**

**#12.** 'brittle diabetes':ab,ti OR 'brittle diabetes mellitus':ab,ti OR 'diabetes mellitus type 1':ab,ti OR 'diabetes mellitus type i':ab,ti OR 'diabetes mellitus, brittle':ab,ti OR 'diabetes mellitus, insulin dependent':ab,ti OR 'diabetes mellitus, insulin-dependent':ab,ti OR 'diabetes mellitus, juvenile onset':ab,ti OR 'diabetes mellitus, type 1':ab,ti OR 'diabetes mellitus, type i':ab,ti OR 'diabetes type 1':ab,ti OR 'diabetes type i':ab,ti OR 'diabetes, juvenile':ab,ti OR 'dm 1':ab,ti OR 'early onset diabetes mellitus':ab,ti OR iddm:ab,ti OR 'insulin dependent diabetes':ab,ti OR 'insulin-dependent diabetes mellitus':ab,ti OR 'juvenile diabetes':ab,ti OR 'juvenile diabetes mellitus':ab,ti OR 'juvenile onset diabetes':ab,ti OR 'juvenile onset diabetes mellitus':ab,ti OR 'ketoacidotic diabetes':ab,ti OR 'labile diabetes mellitus':ab,ti OR 'mckusick 22210':ab,ti OR t1dm:ab,ti OR 'type 1 diabetes':ab,ti OR 'type 1 diabetes mellitus':ab,ti OR 'type i diabetes':ab,ti OR 'type i diabetes mellitus':ab,ti

**#11.** 'insulin dependent diabetes mellitus'/exp

**#10. #5 OR #7 OR #9**

**#9. #3 OR #8**

**#8.**  'echo virus infection':ab,ti OR 'echovirus infections':ab,ti OR 'enteric cytopathogenic human orphan virus infection':ab,ti OR 'infection, echo virus':ab,ti

**#7. #2 OR #6**

**#6.** 'coxsackieviral infection':ab,ti OR 'coxsackievirus infection':ab,ti OR 'coxsackievirus infections':ab,ti OR 'virus infection, coxsackie':ab,ti

**#5. #1 OR #4**

**#4.**  'enteroviral infection':ab,ti OR 'enterovirus infections':ab,ti OR 'infection, enterovirus':ab,ti

**#3.** 'echovirus infection'/exp

**#2.** 'coxsackie virus infection'/exp

**#1**. 'enterovirus infection'/exp
